# *De novo* Transcriptome Assembly and Comparative Analysis Highlight the Primary Mechanism Regulating the Response to Selenium Stimuli in Oats (*Avena sativa* L.)

**DOI:** 10.3389/fpls.2021.625520

**Published:** 2021-06-18

**Authors:** Tao Liu, Xiaoting Liu, Rangrang Zhou, Hong Chen, Huaigang Zhang, Bo Zhang

**Affiliations:** ^1^Key Laboratory of Adaptation and Evolution of Plateau Biota (AEPB), Northwest Institute of Plateau Biology, Chinese Academy of Sciences, Xining, China; ^2^Qinghai Province Key Laboratory of Crop Molecular Breeding, Xining, China; ^3^University of Chinese Academy of Sciences, Beijing, China; ^4^Xining No. 8 Junior High School, Xining, China

**Keywords:** selenium, oats, transcriptome analysis, selenoproteins, selenium metabolism

## Abstract

Selenium is an essential microelement for humans and animals. The specific processing technique of oats can maximize the preservation of its nutrients. In this study, to understand the genetic response of oats in a high-selenium environment, oats were treated with sodium selenate for 24 h, and transcriptome analysis was performed. A total of 211,485,930 clean reads composing 31.30 Gb of clean data were retained for four samples. After assembly, 186,035 unigenes with an average length of 727 bp were generated, and the N50 length was 1,149 bp. Compared with that in the control group, the expression of 7,226 unigenes in the treatment group was upregulated, and 2,618 unigenes were downregulated. Based on the sulfur assimilation pathway and selenocompound metabolic pathway, a total of 27 unigenes related to selenate metabolism were identified. Among them, the expression of both key genes *APS* (ATP sulfurylase) and *APR* (adenosine 5′-phosphosulfate reductase) was upregulated more than 1,000-fold under selenate treatment, while that of *CBL* (cystathionine-β-synthase) was upregulated 3.12-fold. Based on the transcriptome analysis, we suspect that the high-affinity sulfur transporter Sultr1;2 plays a key role in selenate uptake in oats. A preliminary regulatory mechanism explains the oat response to selenate treatment was ultimately proposed based on the transcriptome analysis and previous research.

## Introduction

*Avena sativa* L. (oats) ranks sixth in global cereal production statistics, and oats are grown as multipurpose crops for grain, pasture, and forage or as rotation crops in many parts of the world (Stevens et al., 2004). Cultivated oats are known for their economic value for human nutrition and health care (Singh and Kumar, 2016). Compared to food products such as wheat flour, rice or corn meal, oatmeal retains nearly all the components of the seed, with the micronutrients highly retained (Winkel et al., 2012). Furthermore, oats are rich in protein, fat, vitamins, antioxidants and minerals, which can effectively regulate blood lipids, lower cholesterol and delay the body’s aging (Sadiq Butt et al., 2008). Cultivated oat is an allohexaploid (2n = 6x = 42), consisting of three different genomes with a total size of 12.5 Gbps (Bekele et al., 2018). With the development of next generation sequencing technologies, RNA sequencing (RNA-Seq) could provide an economic and convenient approach for discovering new genes and studying the molecular mechanism on plant abiotic stress, plant development, and diseases resistance (Margulies et al., 2005; Kushwaha et al., 2019). Cheng et al. (2020) found the mechanism of the changes of energy production and AsA-GSH cycle in oat embryos during seed aging by using RNA-seq. Zhang et al. (2021) compared the transcriptome data of an oat male sterile material and a fertile material at different developmental stages, and the results revealed the dynamic expression profile of pollen abortion male sterile and male fertile in oats firstly. It is worth mentioning that PepsiCo and Corteva Agriscience announced the first-ever sequencing of the full oat genome and proposed a new chromosome nomenclature in 2020^1^. This work will greatly promote the vigorous development of the oat industry and accelerate the analysis of various mechanisms in oats while conducive to cultivating oat varieties with improved sustainability, taste and nutrition.

Selenium (Se) is an essential micronutrient for humans and animals (Hatfield and Gladyshev, 2002). It is a key component of more than 25 mammalian selenoenzymes or selenoproteins with important biological functions (Kryukov et al., 2003; Rayman, 2012). Approximately 15% of the global population is affected by Se deficiency due to the consumption of food crops with low Se concentrations (White and Broadley, 2009). For humans, a lack of selenium will lead to a weakened immune system, hypothyroidism, Keshan disease, Kaschin-Beck disease, hair loss, male infertility and so on (Rayman, 2012; White, 2016). In China, one of the 40 countries whose population is deficient in Se, more than 105 million people face adverse health impacts due to Se deficiency (Xu et al., 2012; Dinh et al., 2018). Selenium resources are unevenly distributed in the global environment, and selenium intake for many people is significantly lower than the national recommended standard (Winkel et al., 2012). The eastern region (Ping’an-Ledu area) of Qinghai Province is a selenium-rich area (Zhang et al., 2012; Liu et al., 2014). The humid, cool, and long-day characteristics of this region are very suitable for oat growth. Se biofortification of food crops by means of Se fertilization or breeding Se-enriched cultivars serve as powerful approaches to reduce Se deficiency (Broadley et al., 2007; Hussain et al., 2010; Kumar et al., 2011; Joy et al., 2015). Elucidating the genetic basis of selenium accumulation and metabolism is important for crop biofortification improvement.

In the present study, a comparison of the gene expression profiles of oat seedlings cultured in Hoagland media supplemented with selenium with those cultured in normal Hoagland solution was expected to reflect the metabolic changes resulting in Se accumulation in oats. The final goal of this study was to identify selenium-related genes and to elucidate the relevant metabolic pathways in oats. The assembled and annotated transcriptome will help to understand the genetic basis of selenium uptake, assimilation, transportation and accumulation in oats.

## Materials and Methods

### Plant Material and Se Treatments

*Avena sativa* L. cv. *Jiayan 2* (*Jiayan 2* for short) was selected in this research. This cultivar is a hulled oat widely grown in Qinghai Province, and it produces high yields, displays good quality and is strongly reproducible. The seeds were first soaked in double distilled water (ddH_2_O) for 24 h and then germinated in sterilized petri dishes with moist filter paper at a constant temperature (25°C) in darkness (Wu et al., 2019). Five days later, the seedlings were transplanted to a new container containing 1/2-strength Hoagland’s nutrient solution, under daily conditions of 25°C and a 16/8 h photoperiod (light/dark). Half of the 2-week-old seedlings were treated with 20 μM Na_2_SeO_4_ for 24 h, and the other (as a control group) was not treated. Both the control and treatment groups included two biological replications. The roots of all samples were taken, placed into corresponding numbered centrifuge tubes, immediately frozen in liquid nitrogen, and then stored at −80°C for RNA extraction.

### RNA Extraction, Library Construction, and Sequencing

Total RNA from the roots of oats in the treatment (T) and control groups (CK) was extracted by using a Trizol Plant RNA Extraction Kit according to the user manual (Takara Bio, Japan). The quality of the total RNA was then detected using a 1% agarose gel, and its concentration was measured with a NanoDrop 2000C micro-UV detector (Thermo Fisher Scientific, United States). mRNA was enriched by oligo(dT) beads and fragmented into short fragments according to the manufacturer’s protocol. In conjunction with random primers, mRNA was reverse transcribed into cDNA (Illumina, Inc., San Diego, United States). The cDNA library products were subsequently sequenced in paired-end sequencing technology with read lengths of 150 bp by Gene *Denovo* Biotechnology Co. (Guangzhou, China) by using an Illumina HiSeq^TM^ 4000 instrument.

### Filtering of Sequencing Data and *de novo* Assembly

The raw data contained low-quality sequences, and adapters were obtained from the sequencing machine. Before assembly and analysis, the low-quality reads were removed and trimmed by software Trimmomatic v0.39 (Zheng et al., 2015). The clean reads were then mapped to ribosomal RNA (rRNA) to remove residual rRNA reads and used for further analysis. After purity filtering, the high-quality reads were *de novo* assembled by Trinity, with the default parameters (Grabherr et al., 2011). Sequencing reads were mapped back to the assembled transcripts for assessing the quality of the transcriptome assembly using the Bowtie2 v2.3.4.3 (Li et al., 2009). To evaluate completeness of the *de novo* assembled, BUSCO analysis was performed at default parameters (Kushwaha et al., 2019), and blast was used to validate the assembled contigs with OT3098 genome sequence v1. The longest transcripts of the same genes were screened as unigenes.

### Gene Functional Annotations

With the purpose of acquiring the functional annotations of the unigenes, all the assembled unigenes were searched against the Nr (non-redundant) protein database (Pruitt et al., 2007), SwissProt database (Bairoch and Boeckmann, 1992), KOG (Eukaryotic Orthologous Group) database (Tatusov et al., 2003), and KEGG (Kyoto Encyclopedia of Genes and Genomes) protein pathway database (Kanehisa et al., 2008) by using BLASTX, with an *E*-value < 0.00001 (Altschul et al., 1997).

### Analysis of DEGs

FPKM (fragments per kilobase per million reads) values were calculated and normalized to estimate the unigene expression abundances by software ERANGE (Mortazavi et al., 2008). The differentially expressed genes (DEGs) between the control group and treatment groups of *Jiayan No. 2* were analyzed by the edgeR v3.32.1 (Law et al., 2018). The genes with an FDR (false discovery rate) < 0.001 and an absolute value of the log_2_(ratio) > 1 were considered DEGs (Liu et al., 2017; Song et al., 2017).

To further determine the biological function of the differentially expressed genes, all of them were mapped to the GO (Gene Ontology) database using Blast2GO v4.1.9 (Conesa et al., 2005). The significantly enriched GO terms in the DEGs were analyzed on the online platform “omicshare” with a *p*-value < 0.05^2^. Statistical enrichment of differential expression genes in KEGG pathways with a *p*-value and *Q*-value < 0.05 was performed by KOBAS v3.0 software (Mao et al., 2005).

### qRT-PCR Verification of DEGs

Two microgram purified total RNA from each tissue was reverse transcribed into cDNA using a PrimeScript^TM^ RT reagent kit (Takara Bio Inc.) according to the manufacturer. Then, the cDNA was diluted to 100 ng/μl for relative quantitative real-time RT-PCR analysis. For qRT-PCR validation, a TB Green^®^ Premix Ex Taq^TM^ II kit (Takara Bio Inc.) was used on an ABI ViiA 7 real-time PCR system (Applied Biosystems, United States) with three replicates per sample. The relative expression level of each unigene was calculated by the 2^–ΔΔCT^ method (Livak and Schmittgen, 2001).

## Results

### Illumina Sequencing, Unigene Assembly, and Functional Annotation

To determine the root transcriptome of oat under selenate, a cDNA library was constructed and sequenced. From the four samples, named CK-1 (control check repeat 1), CK-2 (control check repeat 2), T-1 (treated with Na_2_SeO_4_ repeat 1), and T-2 (treated with Na_2_SeO_4_ repeat 2), 216,038,006 raw reads were obtained. After filtering, a total of 211,485,930 clean reads composing 31.30 Gb of clean data were retained, and both the Q20 and Q30 percentages were greater than 95% (Table 1). These data indicated that the sequencing results are of high quality and can be used for further analysis.

**TABLE 1:** Output of sequencing data.

**Sample**	**Total raw reads**	**Total clean reads (%)**	**Total clean nucleotides (bp)**	**Low-quality reads (%)**	**Q20 (%)**	**Q30 (%)**	**GC%**
CK-1	63,782,910	62,371,788 (97.79%)	9,231,820,514	1,111,916 (1.74%)	98.73%	95.97%	54.34%
CK-2	51,002,006	49,918,550 (97.88%)	7,393,057,552	856,134 (1.68%)	98.74%	96.00%	54.39%
T-1	46,531,374	45,581,382 (97.96%)	6,744,793,345	728,406 (1.57%)	98.58%	95.41%	53.67%
T-2	54,721,716	53,614,210 (97.98%)	7,933,128,975	848,136 (1.55%)	98.58%	95.42%	53.98%

**FIGURE 1:**
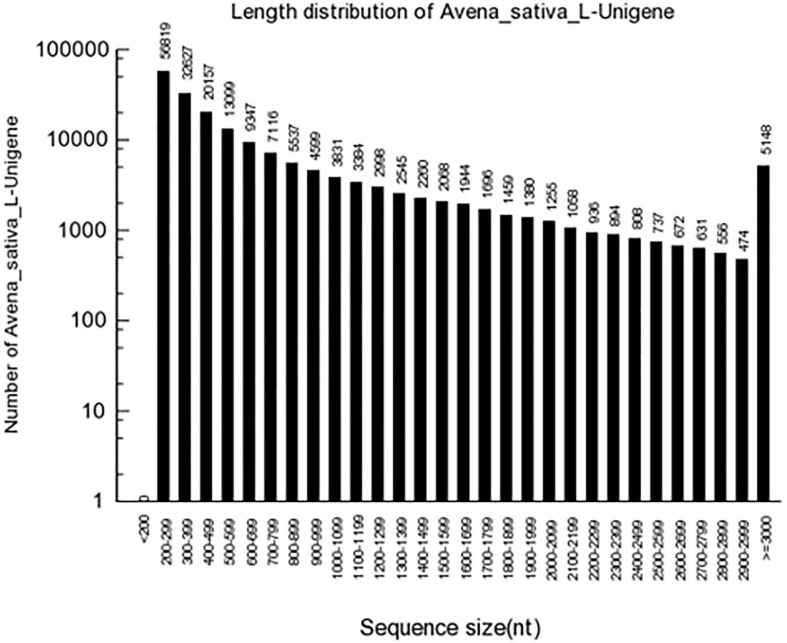
Length distribution of the assembled unigenes.

High-quality clean reads were assembled as contigs and further processed as unigenes with Trinity. A total of 186,035 unigenes with an average length of 727 bp were generated, and the length of N50 was 1,149 bp. Of these, 36,734 (19.75%) unigenes surpassed 1 kb, and 13,169 (7.08%) unigenes were > 2,000 bp (Figure 1). In the transcriptome assembly, BUSCO yielded 2,679 (81.7%) complete BUSCO genes with liliopsida_odb10 database (Supplementary Table 1), and 64.72% contigs could mapped to OT3098 genome v1. The obtained oat transcriptome clean data of 6.7∼9.2 Gb in the present study is less than that predicted oat genome of 12.5Gb. In addition, OT3098 genome v1 could not sufficiently represent its real situation. Above two reasons could explain the lower mapping rate of 64.72%.

**FIGURE 2:**
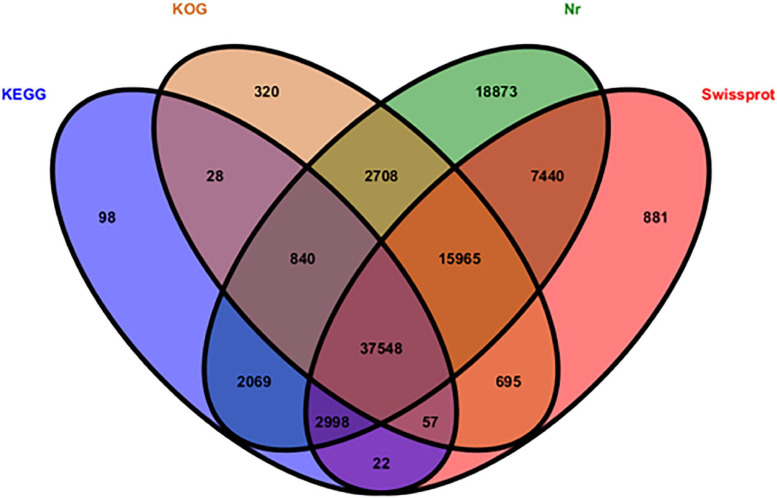
Distribution of functionally annotated unigenes in the KEGG, KOG, Nr, and SwissProt databases.

All 186,035 unigenes were compared with the sequences in several protein databases, including the Nr, SwissProt, KOG, and KEGG databases, by BLAST, wherein 90,542 unigenes showed high homology with sequences in at least one of the above databases (Figure 2). There were 88,441 (97.68%), 65,606 (72.46%), 43,660 (48.22%), and 58,161 (64.24%) unigenes annotated by the Nr, SwissProt, KEGG, and KOG databases, respectively.

**FIGURE 3:**
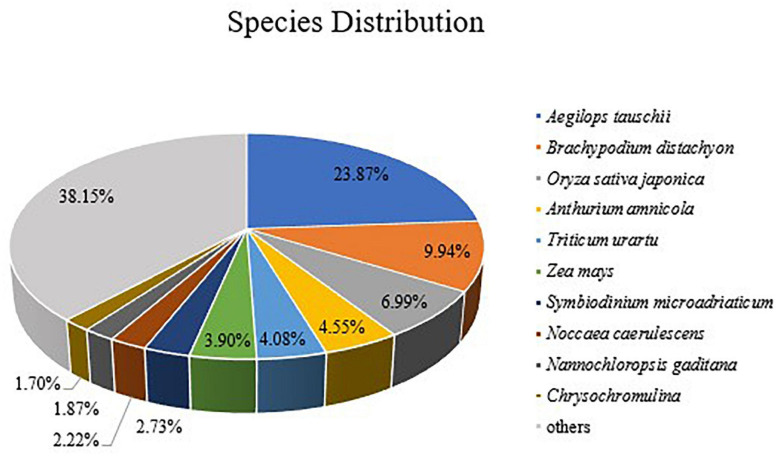
Species distribution of homologous proteins of unigenes. The different color blocks represent different species.

A statistical analysis was performed, and the results showed that 21,111 (23.87%) unigenes matched genes of *Aegilops tauschii*, 8,789 (9.94%) unigenes matched genes of *Brachypodium distachyon*, and 6,183 (6.99%) unigenes matched genes of *Oryza sativa* (Figure 3). We had supposed that more *Avena sativa* L. unigenes would have matched, but that is not the case. The results reflected the truth that little oat genomic information was registered in the NCBI database.

### Analysis of DEGs and GO Classification

**FIGURE 4:**
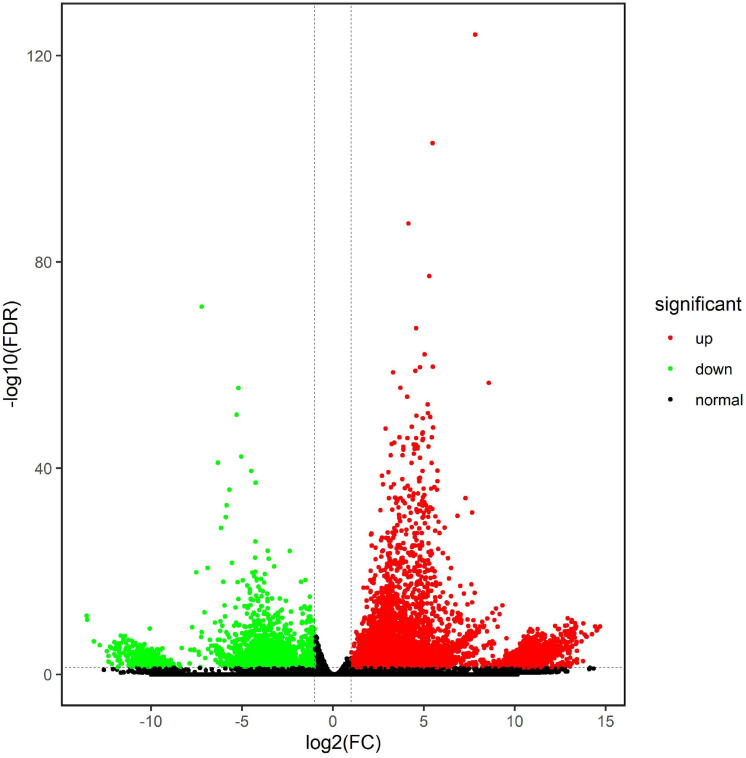
Differentially expressed genes between the CK and treatment groups. The green points represent the downregulated unigenes in the T group, and the red points represent the upregulated unigenes in the T group. The black points indicate that the unigenes have no expression difference between the two groups.

On the basis of the FPKM value of each unigene, the edgeR was used to determine the differentially expressed genes (DEGs) with an FDR (false discovery rate) of < 0.05 and a | log_2_ fold change| of > 1. A total of 9,844 genes were significantly differentially expressed between the CK and Jiayan 2 treatment groups. In total, 7,226 DEGs were upregulated and another 2618 downregulated in the treatment group compared with the control group (Figure 4). The log_2_ fold changes ranged from 1 to 14.70.

**FIGURE 5:**
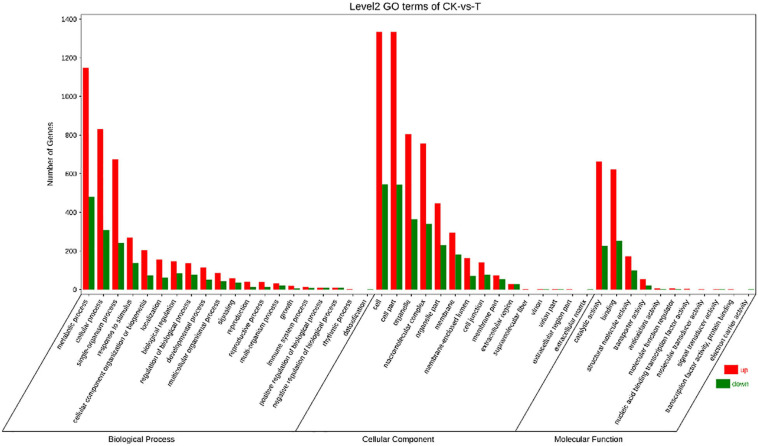
Gene Ontology (GO) classifications of the DEGs between CK and T. Unigenes were assigned to three categories: biological processes, cellular components, and molecular functions.

Based on the Nr database, all DEGs were classified into 46 functional groups (Figure 5). Among these unigenes, 5,671 were related to biological processes, 2,132 were involved in molecular functions, and 7,811 unigenes were associated with cellular components. Metabolic processes (GO:0008152), cellular component processes (GO:0071840), and single-organism processes (GO:0044699) were the dominant groups in the biological process category. In the cellular component category, a high percentage of the DEGs were related to cell (24.04% of the total DEGs) (GO:0005623), cell part (24.03% of the total DEGs) (GO:0044464), and organelle (14.95% of the total DEGs) (GO:0043226). Catalytic activity (GO:0003824), binding (GO:0005488), and structural molecule activity (GO:0005198) were the three top terms in the molecular function ontology. Subsequently, the top20 GO terms in each category were analyzed and found that a large number of genes were enriched in gene expression (GO:0010467), macromolecule metabolic process (GO:0043170), organic substance metabolic process (GO:0071704), cell part (GO:0044464), macromolecular complex (GO:0032991), structural molecular activity (GO:0005198), heterocyclic compound binding (GO:1901363), and organic cyclic compound binding (GO:0097159). Compared with the control group, a large number of genes were enriched in GO terms related to macromolecular compounds in the selenium-treated oats. In other words, selenate is rapidly converted into selenium-containing macromolecular compounds in oats.

**FIGURE 6:**
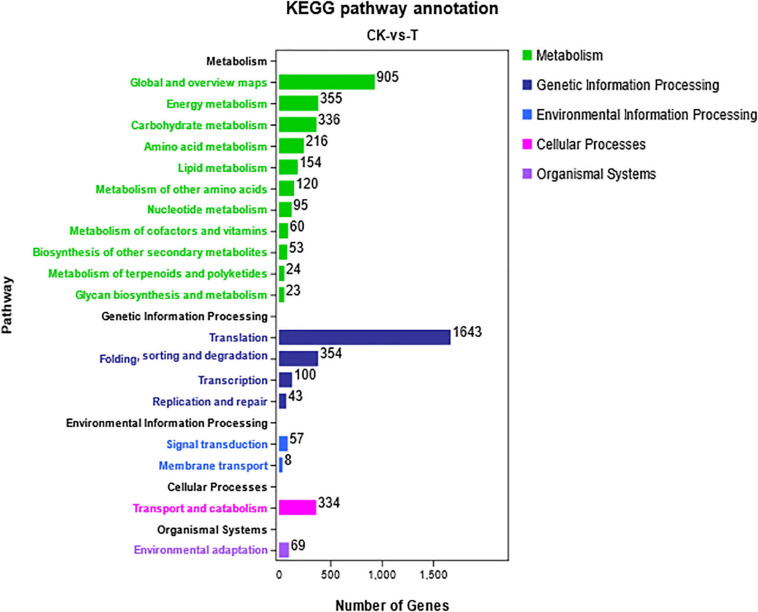
KEGG pathway analysis of differentially expressed unigenes. The DEGs were assigned to five categories: metabolism, genetic information processing, environmental information processing, cellular processes, and organismal systems.

All the DEGs were annotated in five categories with KEGG pathway enrichment analysis (Figure 6). “Global and overview maps,” “transition,” and “signal transport” were enriched in the most unigenes in their respective classifications. Due to the similarity of chemical properties between Se and sulfur, when Se accumulates inside plants, it is incorporated into selenocompounds through the sulfur assimilation pathway (Mostofa et al., 2020). Cysteine and methionine metabolism (ko00270) pertain to amino acid metabolism, and 216 unigenes were associated with this metabolic pathway. Selenocompound metabolism (ko00450) belongs to the category involving the metabolism of other amino acids in the metabolic classification. A total of 120 differentially expressed unigenes were found to participate in the metabolism of other amino acids (Figure 6).

### Expression of Genes Involved in Selenate Metabolism in Oats

**FIGURE 7:**
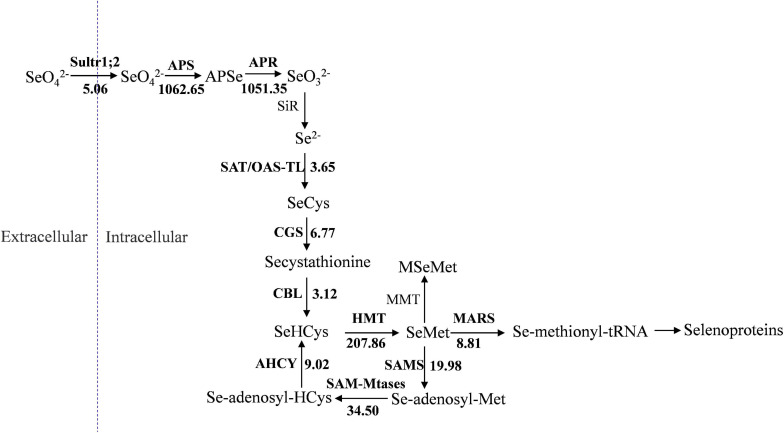
Presumptive expression model of structural genes involved in selenium metabolism in oats. The arrows show the metabolic stream, the short names on the left or above of arrows represent the genes catalyzing the reaction, and the number represents the fold increases of the gene expression after selenium treatment.

Selenocysteine (SeCys), considered the 21st amino acid, was proven to be the active site of all known selenoproteins (Rayman, 2000). Based on the sulfur assimilation pathway and the selenocompound metabolic pathway in higher plants (Ellis and Salt, 2003; Zhu et al., 2009), a total of 27 unigenes related to selenate metabolism were identified by comparison of the gene expression levels in the two groups (Table 2). These unigenes were found to be sequence similarity to the 11 selenate metabolism structural genes: *Sultr1;2* (sulfate transporter), *APS* (ATP sulfurylase), *APR* (adenosine 5′-phosphosulfate reductase), *SAT/OAS-TL* (cysteine synthase complex), *CGS* (cystathionine-γ-synthase), *CBL* (cystathionine-β-synthase), *HMT* (homocysteine methyltransferase), *SAMS* (S-adenosylmethionine synthase), *SAM-Mtase* (S-adenosyl-methionine-dependent methyltransferase), *AHCY* (adenosyl-homocysteinase), and *MARS* (methionyl-tRNA synthetase). El Kassis et al. (2007) conducted selenate resistance studies on 13 *Arabidopsis* mutants and found that sultr1;2 is the only carrier involved in selenate transport in plants. The expression of *Sultr1;2* was 5.06 times higher in the T group than in the CK group, which indicated that selenate was taken by oats and could participate in subsequent metabolic pathways (Figure 7). APS, APR, SiR, and SAT/OAS-TL are key enzymes that catalyze the conversion of inorganic selenium into an organic form (Akbudak and Filiz, 2019; Chen et al., 2019; Harun-Ur-Rashid et al., 2019). Among them, the expression of both *APS* and *APR* increased more than 1,000-fold under selenate treatment, while the expression of *SiR* did not change significantly.

**TABLE 2:** Differential expression analysis of genes related to selenate metabolism in oats.

**Gene**	**KO**	**Gene ID**	**CK_FPKM**	**T_FPKM**	**log2(FC)**	***P*-Value**	**FDR**	**Up/Down**	**Query length (bp)**	**Species**	**Subject length (bp)**	**Alignment length**	**Percent identity**
*Sultr1;2*	k02048	Unigene0013753	64.250	325.408	2.340	4.200E-28	2.710E-25	Up	2,967	*Brachypodium distachyon*	1,682	1,479	87.9%
*APS*	k00958	Unigene0041797	0.001	1.063	10.053	4.010E-05	1.040E-03	Up	1,874	*Triticum aestivum*	1,485	1,355	91.2%
*APR*	K00955	Unigene0079159	0.001	1.051	10.038	4.809E-04	8.333E-03	Up	647	*Anthurium amnicola*	287	195	67.9%
*SAT/OAS-TL*	K13034	Unigene0070348	0.850	3.102	1.868	8.000E-06	2.608E-04	Up	1,754	*Monoraphidium neglectum*	440	290	65.9%
*CGS*	k01739	Unigene0046111	0.945	4.624	2.291	2.988E-04	5.648E-03	Up	1,684	*Brachypodium distachyon*	1,205	1,064	88.3%
		Unigene0077177	1.355	10.950	3.014	2.140E-22	8.450E-20	Up	1,512	*Nannochloropsis gaditana*	580	364	62.8%
*CBL*	k01760	Unigene0091778	1.199	10.742	3.163	2.570E-21	9.090E-19	Up	1,219	*Galdieria sulphuraria*	380	365	96.1%
		Unigene0028653	11.346	27.231	1.263	6.151E-04	1.015E-02	Up	1,420	*Galdieria sulphuraria*	380	364	95.8%
		Unigene0063749	0.811	3.778	2.219	5.600E-06	1.926E-04	Up	1,356	*Nannochloropsis gaditana*	481	431	89.6%
*HMT*	k00548	Unigene0150455	0.015	1.561	6.731	2.680E-07	1.340E-05	Up	1,443	*Anthurium amnicola*	763	463	60.7%
		Unigene0150453	0.001	0.759	9.568	4.534E-04	7.931E-03	Up	796	*Anthurium amnicola*	763	536	34.1%
		Unigene0140031	0.001	1.151	10.168	6.275E-04	1.031E-02	Up	538	*Malus domestica*	302	207	57.9%
*SAMS*	k00789	Unigene0164013	0.079	1.335	4.083	2.471E-03	3.044E-02	Up	811	*Anthurium amnicola*	388	259	66.8%
		Unigene0164011	0.001	3.645	11.832	2.680E-07	1.340E-05	Up	534	*Noccaea caerulescens*	295	168	56.9%
		Unigene0155543	0.001	1.043	10.027	1.153E-03	1.678E-02	Up	484	*Noccaea caerulescens*	295	155	52.5%
		Unigene0056805	0.221	6.244	4.822	1.360E-18	3.850E-16	Up	1,188	*Undaria pinnatifida*	397	385	97.0%
		Unigene0059396	0.522	17.054	5.031	3.170E-37	3.930E-34	Up	1,203	*Undaria pinnatifida*	384	382	99.5%
		Unigene0164014	0.001	2.677	11.386	3.100E-07	1.510E-05	Up	705	*Galdieria sulphuraria*	393	218	55.5%
		Unigene0171862	0.785	0.142	−2.468	1.404E-03	1.960E-02	Down	1,659	*Ectocarpus siliculosus*	397	389	98.0%
*SAM-Mtase*	k01251	Unigene0064410	0.001	3.007	11.554	2.285E-03	2.871E-02	Up	837	*Blastocystis sp. subtype 4*	261	145	55.6%
		Unigene0114896	0.148	2.128	3.848	4.090E-03	4.489E-02	Up	876	*Blastocystis sp. subtype 4*	261	184	70.5%
*AHCY*	k01251	Unigene0162876	2.358	30.985	3.716	4.050E-25	2.040E-22	Up	1,427	*Galdieria sulphuraria*	492	467	94.9%
		Unigene0146505	1.940	0.240	−3.012	3.997E-03	4.409E-02	Down	515	*Anthurium amnicola*	442	341	77.1%
		Unigene0161555	0.001	2.576	11.331	9.210E-06	2.942E-04	Up	649	*Fragilariopsis cylindrus*	481	279	58.0%
		Unigene0030810	0.036	2.360	6.037	6.470E-08	3.730E-06	Up	1,039	*Galdieria sulphuraria*	492	349	70.9%
		Unigene0065022	0.046	3.366	6.185	3.200E-06	1.184E-04	Up	574	*Anthurium amnicola*	431	263	61.0%
*MARS*	k01874	Unigene0006769	0.765	6.738	3.139	1.350E-19	4.150E-17	Up	2,133	*Galdieria sulphuraria*	774	574	74.2%

SeCys is the first molecule for synthesizing selenium-harboring metabolites. Selenomethionine (SeMet) biosynthesis is performed in plants by the sequential action of three enzymes: CGS, CBL, and HMT (Van Huysen et al., 2003; Yang et al., 2004). CGS and CBL, as the rate-limiting enzymes, play crucial roles in the reaction, and their genes are expressed 6.77-fold and 3.12-fold more in the T group than in the CK group, respectively. Three unigenes were found to be homologous to *HMT*, and they had a significant change in expression in oats after Se treatment. Unlike Se-enriched plant species such as onion, garlic, and broccoli, where Se-methylselenocysteine (SeMeSeCys) is a major Se compound, SeMet is the predominant Se form in cereal crop species (Lyons et al., 2005; Zhu et al., 2009). Based on the selenocompound metabolic pathway, it can be concluded that there are multiple catalytic pathways using SeMet as a substrate. MMT (methionine S-methyltransferase) presented no significantly different expression between the CK group and the T group (Figure 7), indicating that oats might not synthesize selenium-methyl selenomethionine (MSeMet) under selenium treatment. By the activity of MARS, SeMet is catalyzed to produce selenoproteins and subsequently participates in plant metabolic regulation.

### Validation of DEGs by qRT-PCR

**FIGURE 8:**
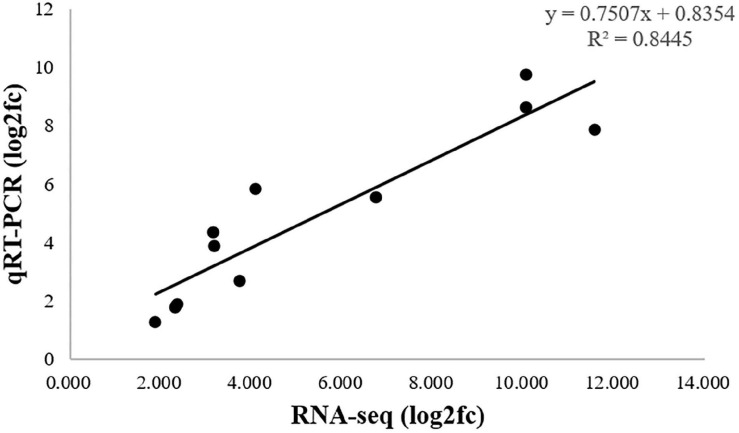
Comparison of expression patterns by RNA-seq and qRT-PCR. The *R*^2^-value is 0.8445.

To validate the RNA-seq results, qRT-PCR was used to quantified analysis the expression of 11 DEGs, *Sultrl1;2*, *APS*, *APR*, *SAT/OAS-TL*, *CGS*, *CBL*, *HMT*, *SAMS*, *SAM-Mtases*, *AHCY*, *MARS*, which were related to selenium metabolism. As shown in Figure 8, the qRT-PCR results (log2fc) of these DEGs were basically consistent with those of RNA-seq data. The correlation coefficient (*R*^2^ = 0.8445) indicated that the DEGs expression pattern obtained by RNA-seq and qRT-qPCR results were in good concordance, which supports the reliability of the RNA-seq data.

## Discussion

Oats are important grain and forage crops worldwide, and oats are grown on 14.1 million hectares, with a grain production that reached 28.3 million metric tons in 2005 (USDA, Foreign Agricultural Service, Commodity production, supply and disposition database). Oatmeal’s unique processing technique allows nutrient elements to be stored in numerous finished products. With more in-depth research on oats, scientists have found that the amino acid composition of oats is more suitable for human nutrition than that of other cereal species and have begun to highly recommend it for human consumption (Zimmer et al., 2019; Zhao et al., 2020). Se from oats is highly bioavailable and regarded as a good dietary source of Se (Yan and Johnson, 2011). Therefore, oats are likely to become one of the dominant food crops in the future. Elucidating the molecular basis and finding key enzymes involved in Se metabolism will be more significant for creating selenium-enriched oat cultivars. Next-generation sequencing technologies have become a powerful technology to illuminate new genes and their involved biochemical pathways in non-model plant species.

In this study, after stimulation with Na_2_SeO_4_ for 24 h, a total of 9,844 DEGs were detected in the CK and T groups after RNA-seq analysis. To elucidate the molecular mechanisms underlying salt tolerance in oat, Wu et al. (2017) utilized 100 mM NaCl to treat hull-less oats and performed a transcriptome analysis; many more (65,000) differentially expressed unigenes were identified. Their results showed that 100 mM NaCl could not only induce Na^+^ and Cl^–^ metabolic pathway genes but also stimulate Na^+^ and Cl^–^ stress-related genes. In our study, 20 μM Na_2_SeO_4_ was used to stimulate related gene expression, which is not a-inducing stress concentration for oats. The differentially expressed unigenes in the present work are mainly focused on selenium uptake, assimilation, transportation, and accumulation. Among them, 7226 DEGs were upregulated in the treatment group, and another 2618 DEGs were downregulated. The absolute log_2_(fold change) values ranged from 1 to 14.70. With the help of functional annotation and KEGG pathway analysis, 27 unigenes related to selenium metabolism were identified, and expression models of related structural genes in oats in response to selenate metabolism were constructed. From the expression model, it could be inferred that MSeMet would not be synthesized and that dimethylselenide (DMSe) would not be synthesized, either (Figure 7); DMSe is one of the major volatile forms of Se produced by plants (Tagmount et al., 2002), and these results are not consistent with those of previous research (Wu et al., 2019). Selenate will be completely converted into selenoproteins after being assimilated by oats and will not form gaseous selenium for release into the environment. Numerous long-term studies have shown that supplemental Se is associated with significant reductions in lung, colorectal and prostate cancers (Ellis and Salt, 2003). Kryukov et al. (2003) found that the human selenoproteome is composed of 25 selenoproteins. They suggested that selenium plays a crucial role in fighting cancer, improving immunity, and maintaining male reproduction because selenoproteins are responsible for biomedical effects in the body.

Because of the similarity of the chemical properties of selenium and sulfur, the absorption of inorganic selenium by plant roots via high-affinity sulfur transporters has been demonstrated by a large number of documents (Sors et al., 2005; Luo et al., 2019). Shinmachi et al. (2010) found that Sultr1;1 and Sultr4;1 transporters in wheat roots played crucial roles in selenate acquisition by S starvation treatment (Shinmachi et al., 2010). Luo et al. (2019) found that *TaSultr1;1*, *TaSultr1;3*, and *TaSultr2;1* gene expression is significantly upregulated after supplying selenide to wheat roots (Luo et al., 2019). In the present study, the expression of *sultrl1;2* was upregulated 5.06-fold under selenate treatment compared with that in the CK group, and the other high-affinity sulfur transporters in oats exhibited no obvious differences. We concluded that *sultrl1;2* played a key role in selenate uptake in oats.

In this research, we utilized the transcriptome to analyze the genetic changes in oats under selenium treatment. The expression pattern of selenium-related structural genes in oats was constructed through KEGG pathway analysis. We speculate that volatile selenium would not be generated in selenium metabolism in oats. To the best of our knowledge, this is the first study to identify the differentially expressed genes related to oat selenium metabolism through combined sequencing in oats. These findings will enrich the understanding of selenium metabolism and lay a foundation for the subsequent identification of key candidate genes for breeding selenium-enriched oat cultivars.

## Data Availability Statement

The original contributions presented in the study are included in the article/Supplementary Material, further inquiries can be directed to the corresponding author/s.

## Author Contributions

HZ and BZ designed the study. TL conducted the experiments and analyzed the data and drafted the manuscript. XL, RZ, HC, and BZ critically reviewed and improved the manuscript. All authors have read and approved the final version of the manuscript.

## Conflict of Interest

The authors declare that the research was conducted in the absence of any commercial or financial relationships that could be construed as a potential conflict of interest. The reviewer ZL declared a shared affiliation, with no collaboration, with the authors to the handling editor at the time of the review.

## Footnotes

1 https://wheat.pw.usda.gov/GG3/node/923

2 https://www.omicshare.com/tools/Home/Soft/gogseasenior
