# Supplementary material for: De novo Transcriptome Assembly and Comparative Analysis Highlight the Primary Mechanism Regulating the Response to Selenium Stimuli in Oats (Avena sativa L.)
Source: Front Plant Sci. 2021 Jun 18;12:625520. doi: 10.3389/fpls.2021.625520 (PMC8249945; doi:10.3389/fpls.2021.625520)
Supplement: Supplementary file 1 [file Table_1.docx]

**Supplementary Table 1|** BUSCO Summary

| C:81.7% [S:78.7%, D:3.0%], F:9.3%, M:9.0%, n:3278 | |
| --- | --- |
| 2679  2581  98  304  295  3278 | Complete BUSCOs (C)  Complete and single-copy BUSCOs (S)  Complete and duplicated BUSCOs (D)  Fragmented BUSCOs (F)  Missing BUSCOs (M)  Total BUSCO groups searched |
